# Peripartum Hysterectomy: Is There Any Difference Between Emergency and Planned Surgeries?

**DOI:** 10.1055/s-0041-1736303

**Published:** 2022-01-29

**Authors:** Tufan Oge, Vehbi Yavuz Tokgoz, Yusuf Cakmak, Melih Velipasaoglu

**Affiliations:** 1 Department of Obstetrics and Gynecology, School of of Medicine, Eskisehir Osmangazi University, Eskisehir, Turkey

**Keywords:** abnormal placentation, emergency, peripartum hysterectomy, planned, uterine atony, placentação anormal, de emergência, histerectomia periparto, planejada, atonia uterina

## Abstract

**Objective** To compare the outcomes of emergency and planned peripartum hysterectomies.

**Methods** The present retrospective cross-sectional study was conducted in two hospitals. Maternal and neonatal outcomes were compared according to emergency and planned peripartum hysterectomies.

**Results** A total of 34,020 deliveries were evaluated retrospectively, and 66 cases of peripartum hysterectomy were analyzed. Of these, 31 were cases of planned surgery, and 35 were cases of emergency surgery. The patients who underwent planned peripartum hysterectomy had a lower rate of blood transfusion (83.9% versus 100%; *p* = 0.014), and higher postoperative hemoglobin levels (9.9 ± 1.3 versus 8.3 ± 1.3; *p* < 0.001) compared with the emergency hysterectomy group. The birth weight was lower, although the appearance, pulse, grimace, activity, and respiration (Apgar) scores were higher in the planned surgery group compared with the emergency cases.

**Conclusion** Planned peripartum hysterectomy with an experienced team results in less need for transfusion and improved neonatal outcomes compared with emergency peripartum hysterectomy.

## Resumo

**Objetivo** Comparar os resultados das histerectomias periparto de emergência e planejada.

**Métodos** Este estudo transversal retrospectivo foi realizado em dois hospitais. Os resultados maternos e neonatais foram comparados de acordo com as histerectomias periparto de emergência e planejada.

**Resultados** Um total de 34.020 partos foram avaliados retrospectivamente, e 66 casos de histerectomia periparto foram analisados. Destes, 31 eram casos de cirurgias planejadas, e 35, cirurgias de emergência. As pacientes que foram submetidas à histerectomia periparto planejada tiveram uma taxa menor de transfusão de sangue (83,9% *versus* 100%; *p* = 0,014), e níveis mais elevados de hemoglobina pós-operatória (9,9 ± 1,3 *versus* 8,3 ± 1,3; *p* < 0,001) em comparação com o grupo de histerectomia de emergência. O peso ao nascer foi menor, embora as pontuações na escala de aparência, frequência cardíaca, irritabilidade reflexa, tônus muscular, e respiração ( *appearance* , *pulse* , *grimace* , *activity* , *and respiration* , Apgar, em inglês) fossem maiores no grupo da cirurgia planejada em comparação com os casos de emergência.

**Conclusão** A histerectomia periparto planejada com uma equipe experiente resulta em menos necessidade de transfusão e melhora os resultados neonatais em relação à histerectomia periparto de emergência.

## Introduction

Peripartum hysterectomy (PPH) is an important surgical procedure that is typically used to prevent maternal mortality from uterine hemorrhage and sepsis. It was first performed at the end of the nineteenth century as a life-saving procedure. 1 The incidence of PPH varies between 0.2 and 6.09 for every thousand deliveries. 2 3 The important risk factors for PPH are age, previous cesarean sections, previous uterine surgery, labor induction, abnormalities in placental invasion, and uterine atony. 4 5 Recent studies 3 6 have reported that the most common indication for PPH was placental invasion anomalies, although uterine atony and uterine rupture were the most frequent reasons to perform PPH in the past. 7 8 The increasing trend in cesarean sections might change the indications in favor of anomalies in placental invasion. 9 Most PPH procedures are performed in an unplanned or emergency situation to prevent life-threatening hemorrhage after unsuccessful conservative approaches such as prostaglandins, tamponade, and compression sutures within 24 hour of a delivery. The morbidity or mortality rates increase with unprepared conditions such as lack of surgical experience and insufficient blood transfusion. Contrary to that, prenatally diagnosed and planned cesarean hysterectomy provides results in low intraoperative bleeding and complications. 10 It also enables surgeons to prepare safe surgical procedures, to prevent morbidities with no increase in intra-/postoperative complications. 11 The aim of the present study was to compare the intra-, postoperative, and neonatal outcomes of patients who underwent emergency or planned PPHs.

## Methods

The present retrospective study was conducted in the Departments of Obstetrics and Gynecology of two hospitals (one tertiary center, and one government hospital) over a period of 23 years. The study was approved by the Research Ethics Committee of the School of Medicine of Eskisehir Osmangazi University (Ref. No: E.98130–2019/19). All women who underwent PPH were included in the study population. Peripartum hysterectomy was defined as hysterectomy performed after 24 weeks of gestation and at the time, or within 24 hours, of delivery. The data of the patients were collected from the medical records, which were reviewed for maternal characteristics such as age, gravidity, parity, gestational age, previous cesarean delivery, and mode of delivery. The preoperative laboratory parameters and indications for surgery were also recorded. The exclusion criteria were as follows: delivery before 24 weeks of gestation and hysterectomy after 24 hours of delivery. The type of the surgery, the intraoperative and postoperative complications, such as ureteral injury, bladder injury, retroperitoneal hematoma, nerve injury, and vessel injury, were investigated. The transfusion of blood products such as red blood cells and fresh frozen plasma performed during and after surgery were measured. The neonatal outcomes were also evaluated, such as birth weight and appearance, pulse, grimace, activity, and respiration (Apgar) scores. The patients were divided into the emergency and planned PPH groups, and the data were compared according to this categorization. Emergency PPH are performed in cases of uncontrollable bleeding with conservative treatment modalities, such as prostaglandins, oxytocics and baloon tamponade. Emergency hysterectomies are performed especially in cases of uncontrollable bleeding and shock, or in cases of previous hemodynamic or hemostatic restoration. Moreover, any type of vascular control is performed with an emergency hysterectomy if necessary. Planned PPH was defined as planned cesarean hysterectomy generally scheduled between the 34th and 37th weeks of gestation. We scheduled planned PPH with a dedicated team composed of an experienced gynecologic oncologist and a maternal-fetal medicine specialist. A preoperative evaluation was performed to determine the specific markers of abnormal placentation with the use of gray-scale and Doppler ultrasound. We administered antenatal corticosteroids before 34 weeks. We performed a midline vertical incision, and the uterus was incised at the fundus. The uterine incision was closed, and dissection of the retroperitoneum and bladder was carefully performed by an experienced surgical team that included a gynecologic oncologist. As much as possible, total abdominal hysterectomy was the main approach, but subtotal hysterectomy was performed in some cases.

The Statistical Package for the Social Sciences (SPSS, SPSS, Inc., Chicago, IL, United States) software, version 15.0, was used to analyze the data. Demographic parameters and clinical outcomes were analyzed with mean ± standard deviation (SD) and median values. The Kolmogorov–Smirnov normality test was used to evalute the distribution of the parameters. Normally distributed data were analyzed by using independent samples *t* -test. The Mann-Whitney U test was used to compare the non-parametric continuous and categorical data. The percentages were compared with the Pearson Chi-squared test or the Fisher Exact test. Values of *p* < 0.05 were considered statistically significant.

## Results

There were 34,020 deliveries during the study period. A total of 66 PPHs were performed, with an incidence of 1.9 for every thousand deliveries, and all cases of PPH were analyzed. The mean age of the patients was 31.3 ± 5.5 years. The gravidity ranged from 1 to 12, with a mean of 3.9 ± 2.4. The average gestational age was 35.7 ± 3.7 weeks. Of these 66 patients, 14 (21.2%) women delivered vaginally, and 52 (78.8%) women underwent cesarean sections. Half of the patients ( *n* = 33; 50%) had at least 1 previous cesarean section. The most common indications for PPH among the sample were placenta accreta ( *n* = 26; 39.4%) and uterine atony ( *n* = 20; 30.3%). Overall, 24 (36.4%) patients underwent subtotal abdominal hsyterectomy, and 42 (63.6%) patients, total abdominal hysterectomy. Planned PPHs were performed in 31 (47%) patients, while emergency PPHs were performed in 35 (53%) cases. Table 1 summarizes the demographic and clinical parameters of the emergency and planned PPH groups. The mean gestational age was significantly lower in the planned PPH group ( *p* = 0.002). Moreover, more than 90% ( *n* = 28) of the patients in the planned group delivered after 34 gestational weeks. The indications for PPH among the study groups are shown in Table 2 . Uterine atony was the most common indication in the emergency group, whereas abnormal placentation was the most common indication in the planned group (57.1%, *n* = 20 and 67.7%, *n* = 21 respectively). We compared the blood transfusions, postoperative laboratory values, and intraoperative complications of both groups ( Table 3 ). The planned PPH group required the use of a significantly lower amount of blood products in the intra- and postoperative periods. The postoperative hemoglobin (Hb) and the differences in pre- and postoperative Hb values were also significantly different between the study groups. The complication rates were similar in both groups. The duration of the hospital stay was shorter in the planned group, but it did not reach statistical significance. Table 4 shows that the neonatal outcomes were significantly different between the groups. The mean birth weight was significantly lower in the planned group, and it might be related to the earlier gestational week at the time of the surgery. Although we have demonstrated the lower birth weight in the planned group, the Apgar scores of this group were significantly better than those of the emergency group ( *p* < 0.01).

**Table 1:** Demographic and preoperative parameters of the groups submitted toemergency and planned hysterectomies

	Emergency hysterectomy ( *n* = 35)	Planned hysterectomy ( *n* = 31)	*p* -value
**Age (years)**	31.9 ± 6.5	30.5 ± 4.1	0.18
**Gravidity**	3.9 ± 2.6	4.1 ± 2.1	0.36
**Parity**	2.9 ± 2.6	2.6 ± 1.8	0.94
**Mean gestational age (weeks)**	36.3 ± 4.9	35.2 ± 1.8	0.002
**Gestational age (weeks) – n(%)**			
< 28	2(6.3)	0(0)	0.169
28–34	5(15.6)	3(9.7)	
> 34	25(78.1)	28(90.3)	
**Mode of delivery – n(%)**			
Vaginal delivery	14(40)	0(0)	0.001
Cesarean delivery	21(60)	31(100)	
**Previous cesarean section – n(%)**	7(20.0)	26(83.9)	0.001
**Preoperative hemoglobin (g/dL)**	10.1 ± 2.2	10.8 ± 1.2	0.10
**Preoperative hematocrit (%)**	32.1 ± 5.8	32.4 ± 3.0	0.84
** Preoperative platelet count (x10 ^9^ /L) **	222.0 ± 73.6	203.2 ± 58.5	0.30

**Table 2:** Indications for peripartum hysterectomy

	Emergency hysterectomy ( *n* = 35)	Planned hysterectomy ( *n* = 31)	Overall ( *n* = 66)
**Uterine atony – n(%)**	20(57.1)	0(0)	20(30.3)
**Uterine rupture – n(%)**	9(25.7)	0(0)	9(13.6)
**Placenta previa – n(%)**	1(2.9)	2(6.5)	3(4.5)
**Placenta accreta – n(%)**	5(14.3)	21(67.7)	26(39.4)
**Placenta percreta – n(%)**	0(0)	8(25.8)	8(12.1)

**Table 3:** Intra- and postoperative outcomes of the patients submitted to emergency and planned hysterectomies

	Emergency hysterectomy ( *n* = 35)	Planned hysterectomy ( *n* = 31)	*p* -value
Red blood cell transfusion – n(%)	35(100)	26(83.9)	0.014
Number of red blood cell transfusions (units)	6.0 ± 5.0	3.9 ± 3.7	0.06
Fresh frozen plasma transfusion – n(%)	34(97.1)	23(74.2)	0.007
Number of fresh frozen plasma transfusions (units)	5.9 ± 5.4	2.5 ± 2.1	0.001
Postoperative hemoglobin (g/dL)	8.3 ± 1.3	9.9 ± 1.3	< 0.001
Postoperative platelet count (x10 ^9^ /L)	119.0 ± 55.8	158.3 ± 44.3	0.12
Difference between pre- and postoperative hemoglobin (g/dL)	1.7 ± 1.5	0.9 ± 0.7	0.03
Difference between pre- and postoperative hemoglobin (after transfusions; g/dL)	7.8 ± 5.9	4.8 ± 4.6	0.02
Vessel injury – n(%)	0(0)	0(0)	1
Nerve injury – n(%)	1(3)	0(0)	0.39
Retroperitoneal hematoma – n(%)	1(3)	0(0)	0.40
Bladder injury – n(%)	4(11)	14(45)	0.38
Ureteral injury – n(%)	0(0)	1(3)	0.39
Duration of hospital stay (days)	8.2 ± 5.9	6.9 ± 3.5	0.57

**Table 4:** Neonatal outcomes of the sample

	Emergency hysterectomy ( *n* = 35)	Planned hysterectomy ( *n* = 31)	*p* -value
**Birth weight (g)**	3041 ± 1186	2564 ± 491	0.003
**Apgar scores**			
1 minute	4.9 ± 3.4	7.5 ± 1.9	0.001
5 minutes	6.5 ± 3.9	9.2 ± 1.1	0.006

## Discussion

The present study showed that the most common indication for PPH was placenta accreata, a subgroup of placental invasion anomalies. The planned PPHs resulted in a lower rate of morbidities and better neonatal outcomes compared with the emergency procedures, which, in turn, required a greater amount of blood products.

The incidence of PPH varies widely. In a large-scaled meta-analysis, 6 the incidence found was of 0.9 for every thousand deliveries. A retrospective cohort study 12 from Pakistan showed a higher incidence, of 4.01 for every thousand deliveries. We have also observed PPH with an incidence of 1.7 for every thousand deliveries in a previous study from our tertiary center. 13 There are several studies that have assessed PPHs, and the incidence may change among countries and centers depending on whether they have sufficient antenatal care for pregnancies. In some studies 7 14 15 16 17 from Turkey, the incidence of PPH was established between 0.3 and 5.38 for every thousand deliveries. Sharma et al. 3 found a much higher incidence, of 6.9 for every thousand deliveries. The incidence found in the present study was similar to those found in previous studies, and in accordance with other Turkish studies. 11 13 14 21 23 In the past, the most common indication used to be uterine atony. 7 8 However, recently, the main indication has been shifted from uterine atony to abnormal placentation. 18 The rising rates of cesarean delivery may result in placental pathologies, increasing the rates of PPH. 6 19 20 21 In a study published in 2016, van den Akker et al. 6 evaluated ∼ 8 million deliveries, and reported that placental abnormalities were the most common indication for PPH, followed by uterine atony. In a recent study, Kazi 12 found that emergency PPH was performed in cases of hemorrhage primarily due to uterine atony. Senturk et al. 17 suggested that the incidence of PPH was higher in Eastern Turkey, and the main indication was uterine atony and rupture. The increasing use of uterotonics and rate of cesarean sections may explain the shift on the main indication for PPH from uterine atony to abnormal invasive placentation. 3 6 19 22 23 24 Morbidly adherent placenta has gained prominent as an indication, especially in planned cesarean PPH. 3 Briery et al. 11 reported that uterine atony was the indication for emergency PPH in over half of the patients, and placenta accreta was the second most frequent indication. In a retrospective study, Sharma et al. 3 showed that placenta accreta was observed in all of the elective PPH patients. We found similar results in accordance to the recent literature. 3 6 11 12 17 The rate of cesarean sections has increased over the years; in the present study, it was of 63.6%. Therefore, placental abnormalities were present in 56% of patients. We have also determined that the indication for PPH was only abnormal placental pathologies in the planned group. We have performed total abdominal hysterectomy in 63.6% ( *n* = 42) of the patients, with no significant differences between both study groups. Subtotal hysterectomy is more desirable for surgeons, because removal of the cervix may be difficult due to possible dilation in cases of PPH. As aforementioned, total abdominal hysterectomy was performed more frequently in the present study. Some studies 7 20 have demonstrated that subtotal abdominal hysterectomy is more suitable, especially in cases of abnormalities in placental invasion, and the morbidity was lower than that of cases of total abdominal hysterectomy. However, some researchers 5 8 have suggested the performance of total abdominal hysterectomy if the patient is in good condition, and they have indicated that this procedure should be considered to prevent hemorrhage from the cervix.

Studies 8 11 have established that intraoperative bleeding is higher in cases of emergency PPH compared with scheduled cases. In a recent prospective-cohort study, Seoud et al. 25 have observed lower volumes of intraoperative bleeding in the elective surgery group, and they have also found that a lower amount of blood products were transfused in the elective cases. In parallel with the higher blood loss, there is a higher amount of transfused blood products in PPH. In the present study, we observed that all of the patients in the emergency group received red blood cell transfusions, and transfusions were necessary in 83.9% ( *n* = 26) of the planned surgery group ( *p* = 0.014). We have also determined that lower volumes of fresh frozen plasma transfusion were required in the planned group. Wei et al. 26 reported a rate of 95% of red blood cell transfusion, and Sak et al. 27 reported a rate of 62.2% among placenta accreta patients. Briery et al. 11 compared the rates of transfusion of red blood cells between patients undergoning emergency and planned cesarean hysterectomies, and they observed rates of 66% and 33%, with a mean of 4.5 and 1.6 of transfused units respectively ( *p* < 0.05). Seoud et al., 25 in their prospective-cohort study, also found that elective surgery was associated with lower rates of blood transfusion compared with emergency surgery. 25 In another retrospective study, 3 the authors reported lower postoperative Hb values in the emergency surgery group compared with the planned group, but without statistical significance (7.8 ± 1.6 versus 8.9 ± 2.2 respectively; *p* = 0.08). In the present study, we have also found significantly lower levels of Hb in the emergency group. The transfused units of red blood cells and fresh frozen plasma were higher in the emergency group. Similar to our study, Seoud et al. 25 established that the transfusion rate and mean transfused units were higher in the emergency cases. We have also analyzed the difference between preoperative and postoperative Hb levels, and found lower differences in the planned cases compared with emergency cases. A higher rate of complications is expected in the emergency PPH group. Bladder injury, which is the most common complication, ranges from 3% to 20% in several studies. 3 7 12 17 23 In the present study, the incidence of bladder injury (27.2%, *n* = 18) was higher than that reported in the literature, and the planned group had a higher rate of bladder injury than emergency group, but this was not statistically significant. We believe that the higher rate may be related to the higher incidence of abnormal placental invasion in the planned group. Briery et al. 11 reported a higher incidence of postoperative complications in the group submitted to emergency cesarean hysterectomy. Two studies 11 25 have established that the length of the hospital stay was similar between the two groups, but Pettit et al. 28 reported a shorter hospital stay in the planned group. We have also observed a slightly longer hospital stay in the emergency group, which was not statistically significant, and was similar to the literature findings.

Neonatal outcomes are important in PPHs. In emergency procedures, these outcomes may be affected negatively, so we can improve the neonatal outcomes by performing planned PPHs in selected patients with proper timing. Seoud et al. 25 reported similar birth weight and Apgar scores among elective and emergency cases. Pettit et al. 28 also compared the neonatal outcomes and reported similar Apgar scores for both groups. Otherwise, they found later gestational weeks and higher birth weights in the planned group. Briery et al. 11 reported that the planned group had later gestational weeks, and higher fetal birth weight and Apgar scores compared with the emergency group, which were not statistically significant. On the contrary, we have observed significantly later gestational weeks and higher birth weight in the emergency group. We have also observed significantly higher Apgar scores in the planned group, although their gestational weeks were later, and the birth weight, lower than those of the emergency group. A possible explanation for that may be administration of antenatal corticosteroids to all of the patients in the planned group prior to delivery.

Emergency PPH is a life-saving procedure, but it results in some postoperative problems. Thus, planned PPH may improve the maternal and neonatal outcomes and decrease the complication rates. The prenatal diagnosis of abnormal placental invasion becomes significant for the performance scheduled surgery. One third of the cases of placental accreta diagnosed prenatally still delivered in an unplanned manner. 28 We think that it is not possible to completely avoid emergency cases. The ideal delivery time for cases of suspected abnormal placentation is still controversial. There were more optimal outcomes regarding the cases of placenta accreta with delivery at the 34th gestational week. 29 The American College of Obstetricians and Gynecologists (ACOG) recently recommended delivery at 34 weeks to 35 weeks and 6 days, especially in cases of suspected placenta accreta. 30 The ACOG also suggests performing the deliveries in cases of placenta accreta with an expert team in a tertiary center. 10

The main limitation of the present study was its retrospective nature. The data of the study population covers a very wide time interval, so cases of uterine atony were more present in older data, and cases of placental pathologies were more prominent in the more recent data. This might establish a selection bias for the present study. However, in the present study, we have comprehensively compared emergency and planned PPHs, and the size of the sample was not large enough according to previous studies. 15 16 28 One of the limitations is the lack of information about the level of expertise of the surgeons in both study groups. Another important limitation is that we have compared different indications for emergency and planned surgeries, such as uterine atony and anomalies in placental invasion; since in cases of uterine atony, the uterine anatomy is not distorted compared to placental invasion anomalies such as placenta accreata.

## Conclusion

We have shown that planning the PHP prenatally improved the maternal and neonatal outcomes. The prenatal diagnosis of suspected cases provides some surgical preparations such as ureteral catheter placement during the surgery. According to the aforementioned ACOG recommendations, we make an effort to diagnose the suspected cases prenatally, and we also currently perform planned PHPs from 34 weeks to 35 weeks and 6 days of gestation with an expert multi-disciplinary team. Further prospective studies are needed to investigate the correlation of planned PHP and perinatal outcomes.
